# The evaluation of the *Woman’s Condom* marketing approach: What value did peer-led interpersonal communication add to the promotion of a new female condom in urban Lusaka?

**DOI:** 10.1371/journal.pone.0225832

**Published:** 2019-12-12

**Authors:** Jessie Pinchoff, Christopher B. Boyer, Rachna Nag Chowdhuri, Gina Smith, Namwinga Chintu, Thoai D. Ngo

**Affiliations:** 1 Population Council, New York, NY, United States of America; 2 Innovations for Poverty Action, New York, NY, United States of America; 3 Innovations for Poverty Action, Lusaka, Zambia; 4 Society for Family Health, Lusaka, Zambia; Boston University School of Public Health, UNITED STATES

## Abstract

During a mass media campaign accompanying the launch of the *Maximum Diva* Woman’s Condom (WC) in Lusaka, Zambia, a cluster-randomized evaluation was implemented to measure the added impact of a peer-led interpersonal communication (IPC) intervention on the awareness and uptake of the new female condom (FC). The WC and mass media campaign were introduced simultaneously in 40 urban wards in April 2016; half of the wards were randomly assigned to the treatment (IPC intervention) with cross-sectional surveys conducted before (n = 2,364) and one year after (n = 2,430) the start of the intervention. A pre-specified intention-to-treat (ITT) analysis measured the impact of randomization to IPC at the community level. In adjusted ITT models, there were no statistically significant differences between intervention and control groups. Due to significant implementation challenges, we also conducted exploratory secondary analyses to estimate effects among those who attended an IPC event (n = 66) using instrumental variable and inverse probability weighting analyses. In addition to increases in FC identification (IPC attendees had higher reported use of any condom, improved perceptions of FC’s, and were more likely to have discussed contraceptive use with their partner as compared to non-attendees). The introduction of a new FC product combined with an IPC intervention significantly increased general knowledge and awareness in the community as compared to media alone, but did not lead to detectable community level impacts on other primary outcomes of interest. Observational evidence from our study suggests that IPC attendance is associated with increased use and negotiation. Future studies should explore the intensity and duration of IPC programming necessary to achieve detectable community level impacts on behavior.

Trial Registration: AEARCTR-0000899

## Background

In low and middle income countries, 214 million women experience an unmet need for family planning[1] and lack contraceptive choices that meet their different needs, particularly where they are at high risk for STIs such as HIV and may lack control over sexual and reproductive decisions due to gender inequity. The female condom (FC) may fill this gap by offering a female initiated method that is effective and provides dual protection. However, FCs represent only 0.8% of the total condoms distributed by donor nations[2]. Historically, the FC has suffered from low demand, due to high cost, inadequate promotion and introduction by programs, and limited acceptability. This low acceptability of the FC includes pervasive male partner objection[3, 4]. However, studies suggest levels of protected sex increase when FCs are added to the available method mix[5–9]. Zambia is an important context for new contraceptive options, as the population experiences both a high unmet need for family planning (27.1%) and an HIV prevalence of 18% in urban areas[10]. To ensure uptake and use of the FC, there must be proactive and well-planned strategies to integrate it into the available mix of contraceptive methods[11].

The Expanding Effective Contraceptive Options (EECO) project supports the introduction of new voluntary contraceptive and dual protection methods, with the goal of addressing method-related reasons for non-use among women and girls with unmet need. Through the EECO project, a new FC product called the Woman’s Condom (WC) was launched in Zambia. The WC, developed by PATH through a human-centered design process, features a thin, flexible pouch made of polyurethane film that conforms to the shape of the vagina[12]. Initial user experience and small qualitative studies found that 57% of women [13] and twice as many men preferred the WC over other FC types[12]. The WC is associated with less failure, and was deemed more acceptable by women than previous FC products[12]. Refining these elements (comfort, safety) is critical to improve ease of use; however, for the WC to be adopted at widely, its introduction must be coupled with a clear marketing strategy.

Despite the many advantages of FC products, to date uptake in sub-Saharan Africa has been low, and this is a concern in the planning of the WC rollout. The EECO partnership launched the WC in urban Zambia under the brand name *Maximum Diva*. In contrast with many historical FC initiatives that lacked investment in demand-generation, the *Maximum Diva* introduction strategy included targeted social marketing and peer-led interpersonal communication (IPC). IPC generally refers to small group discussions fostering behavior change; these are often peer-led, well suited for promoting openness and discussion about sensitive or stigmatizing topics such as condom use. The studies evaluating the impact of IPC interventions have yielded inconclusive findings, but research on behavior change interventions highlight the need to target knowledge including demonstrations of new products, attitudes, and norms[2, 14–16]. We hypothesized that mass media plus a targeted IPC intervention focused on the benefits of dual protection through peer-led interactions (including both men and women) would contribute more to growing the overall condom market than would standard mass-media campaigns alone during the critical introduction period, when the WC was still new.

This study is the first cluster randomized impact evaluation designed to explore community-level impacts of an IPC intervention among a general population of young adults. It measures the added impact of a peer-led, small group IPC intervention on knowledge, acceptability and uptake of the new WC product compared with the distribution and mass media campaign alone. Participants were not always able to distinguish in their language between the WC and the general term FC, so our findings refer to knowledge, perceptions and use of the FC generally. However, the WC was the only product available for sale in Zambia, so any FC use reported at endline must refer to the new product. We will refer to FC use since this is an umbrella term that could encompass any FC product, although at endline this likely refers to the WC that was the only FC product available. While IPC as an intervention has been evaluated rigorously, including for condom use, these studies often target high-risk groups (mainly sex workers)[17], and evaluate IPC participation at the individual level. This study is the first to address a very general population of urban, young adults and to explore the community level impact of a program aimed at demand generation for a new FC product.

## Methods

### Evaluation design

A cluster-randomized controlled trial was implemented across 40 urban wards (administrative units) in Lusaka Province, Zambia, comprised of two cross-sectional surveys of young, sexually-active adults (ages 18–24 years): baseline between November 2015 and January 2016 (n = 2,364), and endline one year later between January and April 2017 (n = 2,430). Different participants were surveyed at baseline and endline, none are repeated as the goal of the trial was to determine whether there was community-level impact. All 40 wards received the mass media campaign, with 20 wards randomized to receive the intervention. The hypothesis was that individuals residing in wards that received the additional IPC intervention would have higher awareness, better perceptions, and increased uptake of the WC product compared with individuals in mass media only wards.

To calculate the sample size needed in each trial arm, we considered prevalence of any condom use as the outcome; the contraceptive prevalence rate in urban Zambia is 53%. The aim was to detect a minimum increase of 5% use with 80% power at the 95% significance level. Because the trial was clustered at the ward level, the sample size was doubled to account for intraclass correlation (ICC) within wards, as no existing estimates were available. Lastly, we increased the sample size by 10% to account for potential refusals. The final sample size was 1,157 individuals per arm or about 58 individuals per ward (n = 2,314).

Each participant gave informed, written consent to participate in the study. This evaluation received approval from both the Innovations for Poverty Action Institutional Review Board (IRB) (IRB No. 10854) and Zambia’s Excellence in Research Ethics and Science (ERES) Converge IRB (IRB No. 00005948). The evaluation is registered with the American Economic Association’s registry of randomized trials (AEA ID: AEARCTR-0000899).

### Randomization

Wards were randomly assigned to receive IPC intervention using computer-generated random assignment. Randomization was done within strata defined by ward size and population as well as pooled values of a few baseline covariates deemed likely to be prognostic of outcomes using a 1:1 allocation ratio. Balance across intervention and control wards at baseline was assessed using a joint test in which the random assignment to receive the IPC intervention was regressed on the vector of baseline covariates. We then calculated the F-statistic testing the hypothesis that all coefficients on the baseline covariates were zero and compared it to the null distribution produced by 10,000 permutated ward-level assignments using randomization inference [18]. A p-value was then calculated by estimating the proportion of assignments which yielded F-statistics as large or larger than the true assignment.

### Data collection

Enumeration areas (EAs) were overlaid for each of the 40 urban wards to divide each ward into multiple sub-units. The geographic centroid of each EA was calculated using ArcGIS v10.2 (ESRI, Redlands, CA, USA). Centroid points within 150m of a ward boundary were excluded, to avoid sampling in incorrect wards. For each survey, data collection teams employed a “random walk” sampling technique starting from a randomly selected list of five centroid GPS points per ward. From each centroid, every third house was approached in each direction, 10–12 households per centroid for a total of 58 households per ward. Households approached were recorded as visited, refused to participate, or no one home (after two follow up attempts). One individual was selected per household to participate. Inclusion criteria were being 18–24 years of age, residing in that house for at least 6 months, and being sexually active. Surveyors were trained to conduct the questionnaire in a private setting, usually within the house. Participants received 10-kwacha (1 USD) scratch off cards for mobile phone use as compensation for their participation. The survey administered was approximately 60 minutes long, and asked structured questions regarding demographics, contraceptive knowledge, access and perceptions. The survey was recorded using SurveyCTO (Dobility Inc, Cambridge MA). Endline surveys included an additional module of questions regarding the FC/WC product, marketing campaign and IPC participation. The endline questionnaire is included as supporting information. Perceptions and behaviors were only measured via questionnaire for the analysis.

### Intervention & monitoring

The intervention has been described in detail previously[19]. In brief, the intervention comprised three components defined as follows:

1. Distribution of the WC: The WC was distributed to pharmacies and other outlets such as chemists and stores across all 40 urban wards (intervention and control wards). The WC was the only version of the FC available in Zambia during the intervention period.
2. Mass media campaign: The WC was advertised widely, targeting young adults through radio, billboards, news media, social media, and a mobile website branded under the “Smart Choices” campaign. The media campaign in its various forms was designed to disseminate information about family planning and serve as an interactive forum for young men and women to ask sexual health related questions and generally discuss SRH related topics.
3. Interpersonal communication (IPC): IPC agents were recruited from wards within the intervention arm for local representation and received training and payment. IPC agents set up an IPC point in a high traffic area of each ward selected for the intervention arm for initial demand creation, recruiting young people for smaller group and one-on-one information sessions (as requested by interested young people). The IPC intervention included communication messages focused on how to use the WC and the benefits of male and female condoms for the prevention of unplanned pregnancies. The key components of the IPC intervention included (1) targeting men and women aged 18–24 years, (2) providing content on the importance of contraceptive use with a focus on the benefits of FCs (specifically the WC), (3) demonstrating how to use the WC on a pelvic model, and (4) training on condom use negotiations with a sex partner through theater or role playing. Information was also distributed in the form of pamphlets, and tools used for the IPC sessions included flip charts and tablets. Each IPC session was about one hour long. The material was designed to be standardized across IPC sessions although the format and topics covered did vary slightly based on interest from the participants.

A process evaluation was conducted during the implementation period to monitor the intervention and ensure fidelity to the various components of the project and measure coverage of the IPC intervention. The process evaluation was done to assess the following: (1) availability and distribution of the WC, (2) tracking of the mass media campaign, (3) penetration and reach of the IPC intervention, and (4) acceptability of the WC. First, we implemented a mystery shopper survey in half of the study wards to visit outlets and determine if the WC was available, in stock, and coupled with any advertising such as posters. This was cross-checked with previously collected data regarding distribution to outlets. Second, we monitored aggregated website data to measure traffic to the site, and checked that billboards were still in place. Third, we conducted spot checks of IPC events and shadowing of IPC agents, to monitor recruitment and ensure that all components of the IPC curriculum were routinely covered. Lastly, we conducted 30 focus group discussions to discuss perceptions and awareness of the WC product. Program monitoring occurred throughout the one-year intervention period. The process evaluation data was used to contextualize RCT findings and in particular the focus groups were used to gain a more nuanced understanding of family planning norms and concerns among both men and women ages 18–24 years. The process evaluation also identified the low availability of the WC product and the varied intensity in IPC across intervention wards.

### Statistical analysis

Primary and secondary outcomes of interest were pre-specified in prior to data collection and are described in Table 1. All participants were initially analyzed as randomized (intention to treat) regardless of whether they attended an IPC event. Intention to treat effects were estimated using a least squares regression of the following form:

$$Y_{ijk}=\gamma_{k}+\tau Z_{ijk}+\beta^{t}{\bar{X}}_{ijk}+\lambda^{t}Z_{ijk}{\bar{X}}_{ijk}$$

Where *i*, *j*, and *k* index the individual, ward, and randomization strata respectively, *Z_ijk_* is an indicator of ward-level assignment to the IPC intervention, ${\bar{X}}_{ijk}$ is a vector of mean-centered covariates, and *γ_k_* is a fixed effect for the *k*’th randomization strata. The coefficient *τ* represents the conditional average treatment effect under intention-to-treat. Estimates are presented for both adjusted (with the mean-centered covariates) and unadjusted specifications (without mean-centered covariates) of the model. The covariates included pooled estimates of the outcome at baseline as well as the following post-treatment variables that were believed to be plausibly invariant to the intervention: gender, age, education level, marital status, and employment status Pre-specified sub-group analyses were conducted by gender and marital status but were considered exploratory.

**Table 1:** Outcomes of interest.

Variable	Format	Definition
*Primary Outcomes*		
MC use	Binary	Self-reported; ever, in last 6 months, at most recent sexual intercourse.
FC use (includes WC)	Binary	Self-reported; ever, in last 6 months, at most recent sexual intercourse. Participants were not consistent in identifying the WC (brand name) from FC (general term) so we refer to the FC in our results.
Interest in trying FC	Binary	Self-reported; interest in trying an FC in the future
*Secondary Outcomes*		
Correct knowledge of FC	Binary	Combined variable of self-reported awareness of the FC and correct identification of a picture of an FC
Partner communication	Binary	Self-reported negotiated condom use with most recent sexual partner
MC perceptions	Index	Multiple correspondence analysis to create an index of perceptions of MCs based on selection of true or false questions adapted from a validated WHO tool.[20]
FC perceptions	Index	Multiple correspondence analysis to create an index of perceptions of FCs based on selection of true or false questions adapted from a validated WHO tool.[20]

Due to lower adherence and diffusion of the IPC intervention in the treatment wards than expected as well as spillover contamination in the control wards (i.e. two-sided non-compliance), we conducted two exploratory secondary analyses to estimate the effect of adherence. In the first analysis, we defined wards with at least one person reporting attending an IPC event as “treated” and used the original randomization as an instrument to estimate the effect of IPC among the subset of wards that were treated using two-stage least squares. This analysis estimates the community level effect of adherence. Next we conducted a second analysis to estimate the effect of attending an IPC event among the individuals who reported attending using inverse probability weighting (IPW) [21]. To construct the weights, we used logistic regression to estimate the probability of attending at least one IPC event at endline conditional on the same set of covariates mentioned in the intent-to-treat analysis above. We then estimated the effect of IPC attendance using weighted least squares regression models of the primary outcomes on an indicator of IPC attendance with heteroskedasticity robust standard errors [22]. These models provide an unbiased estimate of the effect of the IPC intervention if everyone in the sample attended compared to if no one attended, under the strong assumption of no unmeasured confounding (i.e., that there was no unobserved covariate that predicted attending and IPC and the outcome).

## Results

### Study population

A total of 4,817 interviews were completed; 2,364 at baseline and 2,430 at endline. At endline, almost three quarters of participants were female (73%); the average age was 21.3 years (standard deviation = 1.9 years); over half were literate (67%); about a third were married (32%); and half already had one or more children (48%). Randomization-based balance tests comparing the composition of treatment and control wards across invariant characteristics at baseline (F = 4.131 p = 0.719) and again at endline (F = 1.132; p = 0.920) imply the sharp null hypothesis that ward composition is the same cannot be rejected at conventional levels (Table 2).

**Table 2:** Characteristics of treatment and control wards, Lusaka, Zambia 2015 (Balance table).

	Baseline			Endline		
	(N = 2364; M = 40)			(N = 2430; M = 40)		
Variable	Treatment	Control	*p*-value	Treatment	Control	*p*-value
*Socio-demographic controls*						
Female [0,1]	0.675	0.704	0.519	0.748	0.719	0.177
Age	21.360	21.275	0.201	21.200	21.353	0.041
Completed some secondary school [0,1]	0.671	0.607	0.134	0.649	0.608	0.380
Completed some post-secondary school [0,1]	0.143	0.143	0.595	0.130	0.140	0.541
Can read and write [0,1]	0.807	0.741	0.320	0.708	0.635	0.425
Married [0,1]	0.327	0.380	0.808	0.290	0.341	0.518
Has children [0,1]	0.477	0.511	0.956	0.458	0.509	0.546
Is currently employed [0,1]	0.217	0.196	0.411	0.198	0.217	0.375
Age at first sex	17.315	17.305	0.614	17.477	17.215	0.040
Lifetime sex partners (n)	3.634	3.386	0.623	3.581	3.303	0.891
Sex partners in last 6 months (n)	1.285	1.277	0.998	1.296	1.296	0.796
Frequency of sex in last month (n)	3.645	3.624	0.433	0.993	1.007	0.708
Has been tested for STIs [0,1]	0.799	0.794	0.497	0.792	0.826	0.570
Has experienced a male condom break [0,1]	0.234	0.189	0.111	0.218	0.230	0.545
Travels at least 30 min to buy contraception [0,1]	0.354	0.408	0.417	0.363	0.360	0.556
*Primary outcomes*						
Ever used female condom [0,1]	0.057	0.050	0.377			
Used female condom in last 6 months [0,1]	0.018	0.025	0.241			
Used female condom at most recent sex [0,1]	0.006	0.016	0.037			
Ever used any condom [0,1]	0.867	0.841	0.209			
Used any condom in last 6 months [0,1]	0.603	0.548	0.444			
Used any condom at most recent sex [0,1]	0.433	0.390	0.674			
Would be willing to try a female condom [0,1]	0.603	0.570	0.387			
*Secondary outcomes*						
Contraceptive knowledge index, z-score	-0.362	-0.366	0.697			
Correctly identifies a female condom [0,1]	0.033	-0.032	0.564			
Male condoms attitudes index, z-score	0.063	-0.063	0.056			
Female condoms attitudes index, z-score	0.531	0.459	0.069			
Discussed contraceptive use, [0,1]	0.631	0.607	0.560			
Observations (*N*)	1177	1187		1204	1226	
Clusters (*M*)	20	20		20	20	
*F*-statistic			4.131			1.132
*p*-value			0.719			0.920

### Intention to treat results

**Fig 1:**
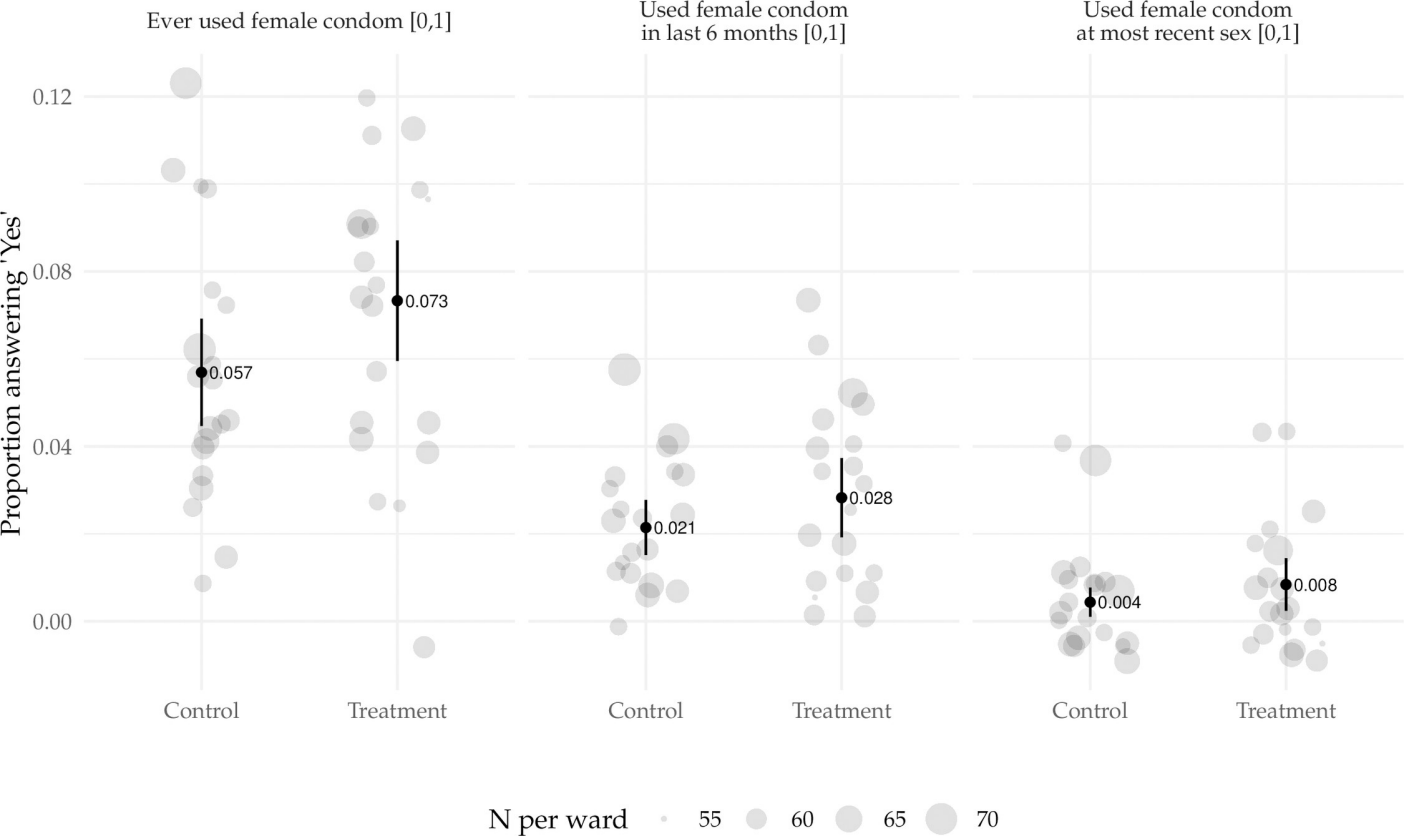
Proportion responding yes for the three primary outcomes (ever used a FC, used FC in last 6 months, used FC at most recent sex) per ward, comparing treatment to control arms.

The proportion in each ward ever using a FC, using an FC in the last 6 months, and using an FC at most recent sex were tabulated by control and treatment arm, highlighting slightly higher proportions for all three of the primary outcomes in treatment wards (Fig 1).

An intention-to-treat (ITT) analysis was conducted comparing wards as randomized. In unadjusted models, intervention wards reported higher rates of FC use and lower rates of male condom use; however none of these results were statistically significant at the pre-specified levels (Table 3). Similar patterns persist in models adjusted for gender, age, education level, marital status, employment status, and pooled estimates of the outcomes at baseline. Conditional on these covariates, the estimated effect of the IPC intervention is a 1.8 percentage point increase in female condom use (*τ* = 0.018, 95% CI [-0.001, 0.035], p = 0.06) which is statistically significant at the 10% level, however it is not significant at the pre-specified 5% level (Table 3).

**Table 3:** Intention to Treat (ITT) effects of IPC Intervention on Primary Outcomes (unadjusted and adjusted).

	Unadjusted			Adjusted		
	*τ*	95% CI	*p*-value	*τ*	95% CI	*p*-value
*Primary outcomes*						
Ever used female condom [0,1]	0.013	(-0.003, 0.030)	0.139	0.018	(0.001, 0.035)	0.061
Used female condom in last 6 months [0,1]	0.006	(-0.005, 0.017)	0.366	0.007	(-0.004, 0.017)	0.315
Used female condom at most recent sex [0,1]	0.001	(-0.007, 0.008)	0.830	0.003	(-0.004, 0.011)	0.358
Ever used any condom [0,1]	-0.016	(-0.051, 0.020)	0.392	-0.015	(-0.045, 0.014)	0.335
Used any condom in last 6 months [0,1]	-0.012	(-0.077, 0.053)	0.724	-0.016	(-0.050, 0.018)	0.417
Used any condom at most recent sex [0,1]	-0.010	(-0.074, 0.055)	0.770	-0.007	(-0.043, 0.028)	0.723
Would be willing to try a female condom [0,1]	0.001	(-0.041, 0.043)	0.961	0.003	(-0.043, 0.049)	0.909
*Secondary outcomes*						
Contraceptive knowledge index, z-score	0.018	(-0.111, 0.147)	0.783	0.009	(-0.073, 0.091)	0.885
Correctly identifies a female condom [0,1]	0.040	(-0.019, 0.099)	0.237	0.038	(-0.007, 0.084)	0.171
Male condoms attitudes index, z-score	-0.007	(-0.149, 0.135)	0.924	-0.039	(-0.145, 0.068)	0.526
Female condoms attitudes index, z-score	0.039	(-0.069, 0.147)	0.522	0.028	(-0.077, 0.133)	0.639
Discussed contraceptive use, [0,1]	-0.008	(-0.070, 0.053)	0.786	-0.013	(-0.069, 0.043)	0.663

**Fig 2:**
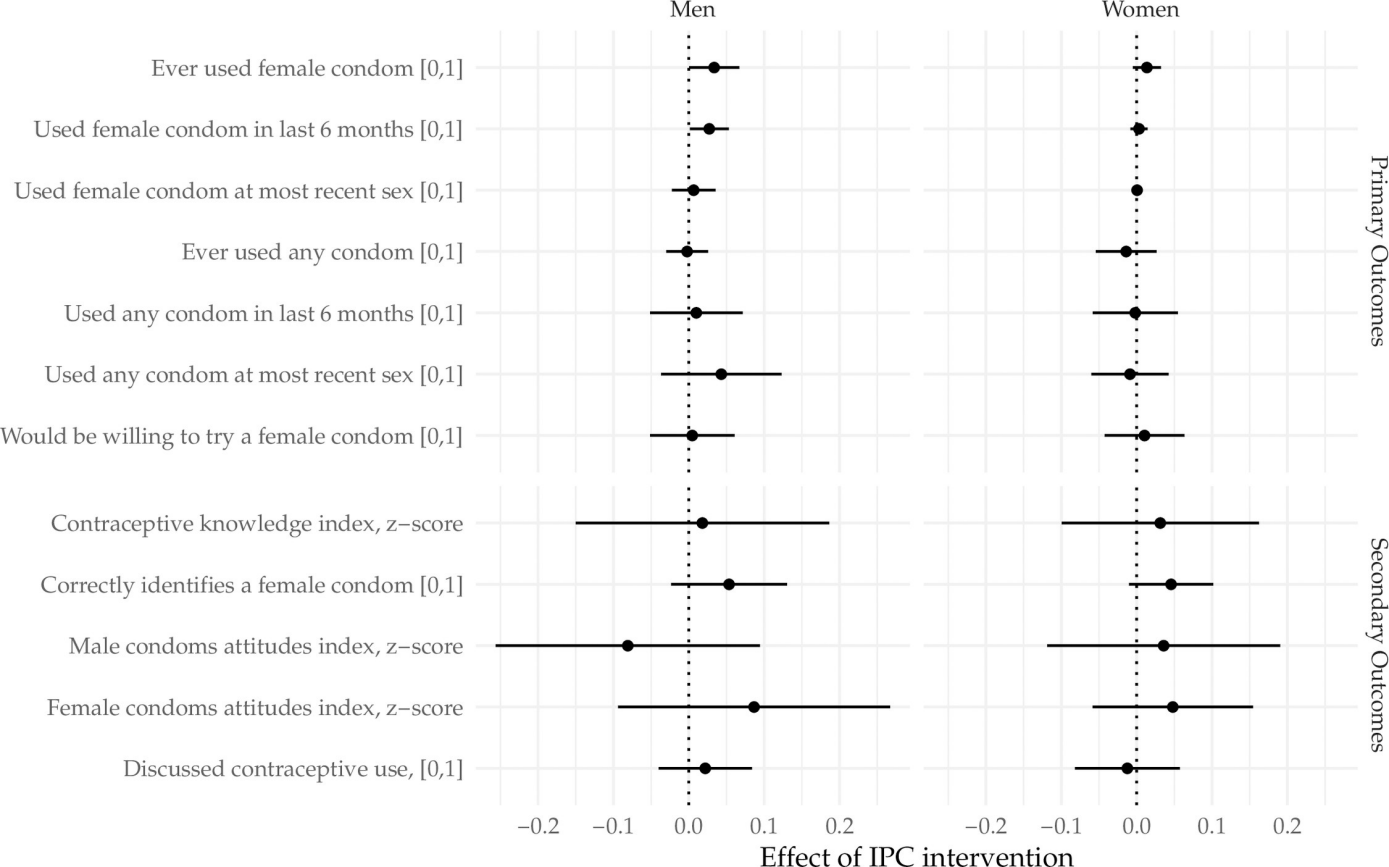
Gender specific effects of the IPC intervention on key primary and secondary outcomes.

**Fig 3:**
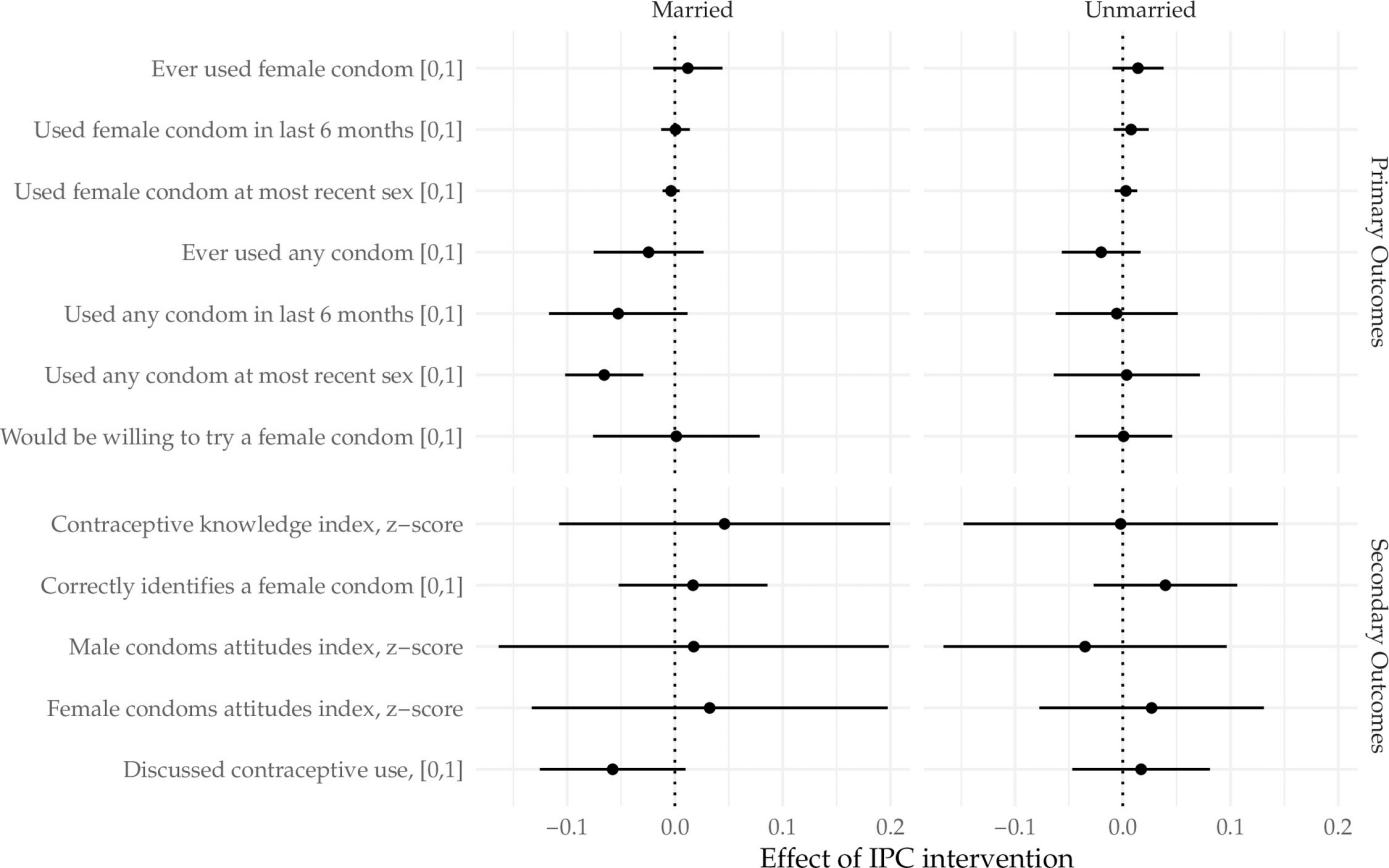
Marital status specific effects of the IPC intervention on key primary and secondary outcomes.

Heterogeneous effects by gender and by marital status were explored. Although there were no statistically significant differences in the effect of IPC intervention between the genders overall, it was noted that among men only, those in IPC intervention wards were slightly more likely to report ever using the FC (p = 0.09), or using the FC in the last 6 months (p = 0.08), however, this was not significantly different from the estimate of the effect among women (p = 0.371 for ever use, p = 0.146 for last 6 months) (Fig 2).We observed significant differences in the effect of randomization to IPC on the probability of discussing contraception by marital status (p = 0.027) and the probability of using male condoms at most recent sex (p = 0.079). Among unmarried respondents, randomization to IPC increased the probability of discussing contraception by 1.7 percentage points (95% CI [-0.05, 0.08]) while randomization to IPC decreased the probability by 5.8 percentage points (95% CI [-0.13, 0.01]) among married respondents (Fig 3). Similarly among married respondents, randomization to IPC decreased the probability of using a male condom at most recent sex by 6.6 percentage points (95% CI [-0.10, -0.03]).

### Monitoring results

**Fig 4:**
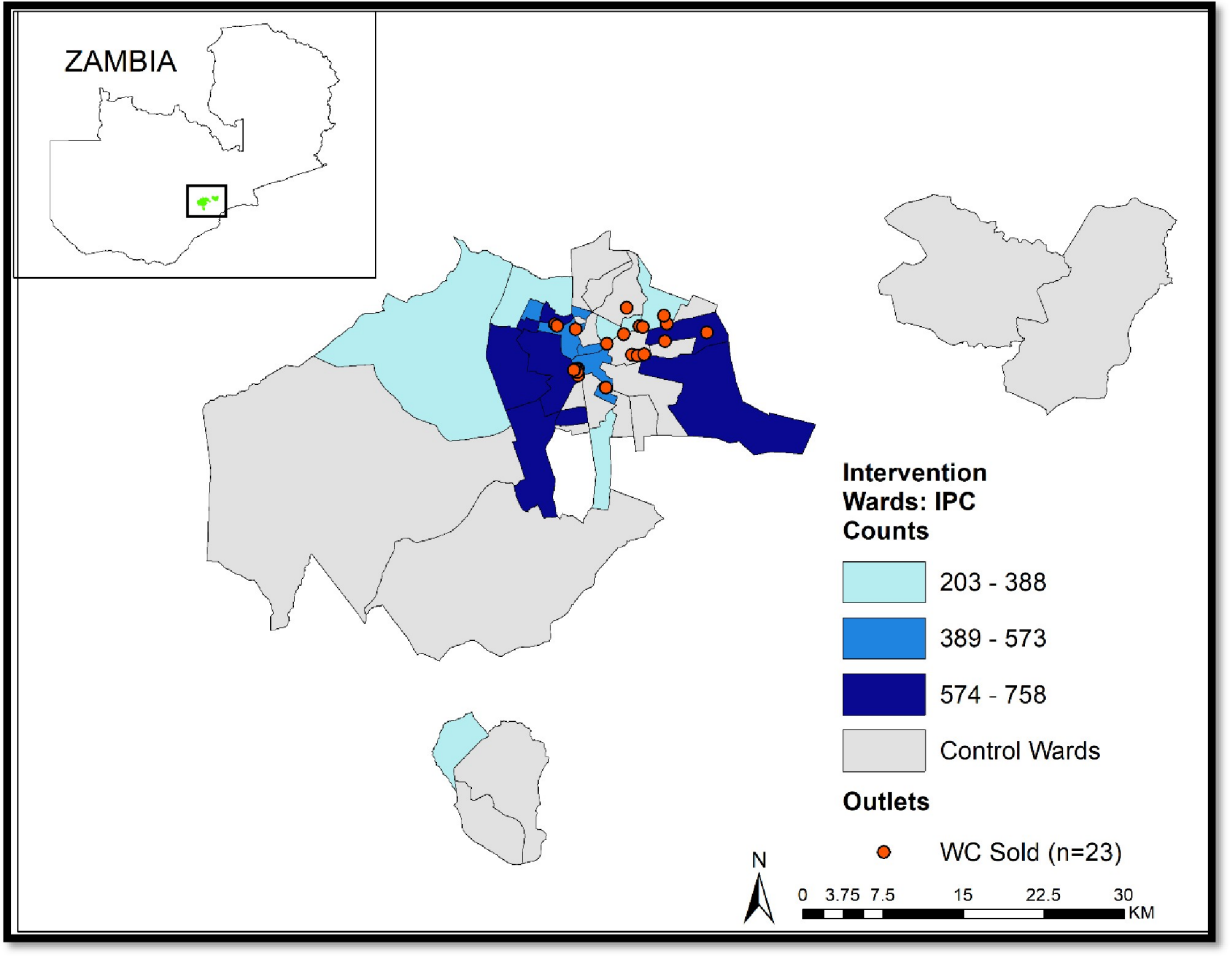
Map of IPC coverage per intervention ward and location of outlets selling the WC (as detected during a mystery shopper activity).

During the one-year intervention period, IPC agents conducted 9,943 IPC sessions that included 34,087 attendees. 55% of attendees were female (n = 18,639). 40% (n = 13,555) were within the target age range, with most in the 25–35 year age range. Mystery shoppers visited 22 of the 40 study wards in which a listing exercise was previously conducted. Of the 532 outlets visited, only 27 (5%) stocked the WC product at the time of the visit. Of the 27 outlets that did stock it, 78% were pharmacies. One quarter of participants reported they were familiar with the FC and the WC specifically, and of those surveyed, only 3% reported attending an IPC session (n = 66). The number of IPC events hosted and the number of outlets identified selling the WC varied by ward (Fig 4).

### Estimated community level adherence results

Due to low levels of people reporting attending an IPC event in treatment wards in the endline (N = 66) as well as implementation challenges and reported spillover in control wards, further exploratory analyses were undertaken to estimate both the community level effect of IPC among those living in a ward in which at least one person reported attending an IPC event (IV analysis) as well as the individual effect of attending an IPC event (IPW analysis). Compared with the ITT results, the IV analysis suggests that community level effect estimates for primary and secondary outcomes are stronger across wards where at least one person at endline reported attending an IPC event; however none of these are statistically significant. In the IPW results, participants who attended IPC were more likely to report using any condom (male or female) (*τ* = 0.105; 95% CI [0.067, 0.143]), and to correctly identify a FC (*τ* = 0.243; 95% CI [0.081, 0.405]) (Table 4). They also had a 0.556 standard deviation increase in the FC attitudes index indicating improved perceptions of FC’s (*τ* = 0.556; 95% CI [0.126, 0.985]) (Table 4). Lastly, those who attended were 16% percentage points more likely to discuss contraceptive use with their partner (*τ* = 0.163; 95% CI [0.081, 0.246]) (Table 4).

**Table 4:** Instrumental variable and inverse probability weighting analyses to assess compliance effects of attending the IPC intervention.

	IV		IPW	
	*τ*	95% CI	*τ*	95% CI
*Primary outcomes*				
Ever used female condom [0,1]	0.064	(-0.028, 0.156)	0.064	(-0.020, 0.148)
Used female condom in last 6 months [0,1]	0.025	(-0.033, 0.082)	0.032	(-0.015, 0.080)
Used female condom at most recent sex [0,1]	0.015	(-0.019, 0.048)	-0.008	(-0.012, -0.004)
Ever used any condom [0,1]	-0.058	(-0.192, 0.077)	0.105	(0.067, 0.143)
Used any condom in last 6 months [0,1]	-0.173	(-0.652, 0.307)	0.060	(-0.130, 0.250)
Used any condom at most recent sex [0,1]	-0.068	(-0.330, 0.195)	-0.071	(-0.242, 0.101)
Would be willing to try a female condom [0,1]	0.025	(-0.225, 0.275)	0.104	(-0.050, 0.259)
*Secondary outcomes*				
Contraceptive knowledge index, z-score	0.948	(-2.454, 4.351)	0.144	(-0.153, 0.441)
Correctly identifies a female condom [0,1]	0.137	(-0.075, 0.350)	0.243	(0.081, 0.405)
Male condoms attitudes index, z-score	0.272	(-1.787, 2.331)	0.135	(-0.155, 0.424)
Female condoms attitudes index, z-score	0.076	(-0.344, 0.496)	0.556	(0.126, 0.985)
Discussed contraceptive use, [0,1]	-0.060	(-0.326, 0.205)	0.163	(0.081, 0.246)

## Discussion

This is the first cluster randomized evaluation of the impact of the WC coupled with IPC at the community level in an urban sub-Saharan African setting, targeting a general young adult population. Our trial demonstrated that after one year, a quarter of participants reported they were familiar with the WC campaign, having seen the mass media or heard about it from a friend or health worker. Although participants were not consistent in terminology between the WC vs a general FC product, the WC was the only version of a FC available on the market and therefore we assumed responses regarding the FC were in reference to the WC. The ITT model did not detect any community level differences between intervention and control groups with regards to awareness of the new FC product or its use. This may be because of intervention challenges (variation in intensity of IPC across intervention wards resulting in low attendance overall, lack of availability of the WC product in outlets, high spillover of those residing in control wards attending IPC events in intervention wards). The IPW analyses of compliance effects highlighted significant increases in reported use of any condom (male or female), correct identification of an FC, improved FC perceptions, and higher reported discussion of contraception with a partner. While these effects were not detected at the community level using the more rigorous randomized design, our compliance effects findings are supported in the literature and warrant further exploration.

IPC for behavior change is promising, suggesting exposure to IPC is associated with knowledge acquisition and acceptability of sensitive topics such as condom use[23]. They may also diffuse messages through social networks and communities to spread information and reinforce condom use norms, increasing acceptability[24]. The IPC curriculum for this study included components identified as critical to uptake by previous studies, for example, that IPC must address attitudinal and cultural barriers[25], include product demonstration[26], and improve partner communication[27]. Uptake of the WC depends on being able to change perceptions, gender relations, and culture to increase acceptability [4]. For example, some studies report the FC is stigmatized as the product is linked with infidelity and commercial sex work[4]. IPC is a potentially useful tool necessary to complement the rollout of an FC product to address norms and contextual factors that act as a barrier to uptake and acceptability. While our ITT model results did not detect an effect, this may suggest that a higher coverage of IPC over more than a one year time period, with higher product availability, is necessary to change perceptions and use and sustain these changes. The lack of findings may suggest that the IPC intervention was not sufficient to change perceptions and use, but may also reflect some of the implementation challenges faced.

Identified implementation challenges regarding IPC attendance rates likely limited the detection of impact on outcomes of interest. Variation in the number of IPC events held per ward resulted in uneven access. Second, although this improved over time, there was some misalignment between survey and IPC populations. Due to the survey protocol, only 18–24 year olds were eligible; however, IPC attendees were slightly older (60% were 25–30 years old), and more gender balanced (53% female IPC attendees vs 70% of survey participants). Thus, the survey results may not have reflected the population of IPC attendees. Third, the one-year implementation period may not have been sufficient to stock the product for a community level impact. Findings from qualitative data collected during the implementation period suggest the product was not commonly for sale, and mystery shopper findings support this as only 5% of randomly visited outlets stocked the product. Lack of availability and access is a major barrier to use that has been identified in many studies [4]. Lastly, IPC coverage may have been too low to detect an effect using this study design. The hypothesis was that IPC would have sufficient saturation and coverage throughout intervention wards, with relatively little spillover to control wards; however, about half of survey participants who attended IPC were from control ward likely because individuals travel freely and IPC agents encouraged bringing friends or partners (regardless of ward of residence). Due to the cross-sectional design, tracking behavior of individual IPC attendees before and after exposure was not possible, but estimated community adherence results from our secondary analysis do suggest an effect.

Our findings can inform future health communication programs, including efforts to promote FCs in urban African settings. Challenges in procurement and distribution of the product over the short implementation period likely limited the ability to detect an effect of our intervention; however, these challenges are often faced in evaluations of field-based programs. To understand these implementation challenges, we collected monitoring and qualitative data to contextualize the evaluation results and understand the realities on the ground, which suggested a positive positioning for the WC product. Study participants reported high interest; in the future, 38% were interested in using a MC (vs 64% interested in using a FC). There is a market for the WC product, but additional research may be necessary to determine: first, the required duration of implementation and coverage of IPC to see diffusion of effects at the community level, and second, to identify whether different segmentation is required. Within the narrow age range of 18–24 years, many participants were married and some already had children–both major factors determining contraceptive preferences. Also, as discussed above, slightly older individuals (24–35 years of age) may be most interested. It is unclear if the slightly older age of IPC attendees reflects these demographic characteristics, or just that they were more likely to be recruited to IPC sessions or more willing to attend, but might indicate the need for a different recruitment strategy.

Overall, findings suggest that within a short implementation period of one year, the new WC with its specific branding of *Maximum Diva* was recognized by a quarter of young adults in Lusaka, and there is strong interest in this product. Although not detected at the community level using a rigorous ITT design, IPC attendance was found to increase awareness of the product among ‘compliers’. In addition, ‘compliers’ in our secondary analysis reported increased use of any condom, improved perceptions of FC’s and increased partner communication regarding contraception. IPC is a useful strategy to change norms and perceptions around FC products, which is critical to increase acceptability and uptake. Adding an FC product such as the WC to the method mix in a country increases the overall proportion of protected sexual acts in a population, making it an important option to make available[4]. Process evaluation and qualitative data collection are important to contextualize evaluation results and improve understanding of implementation challenges faced. Future research should determine the duration, intensity and frequency of the IPC necessary to effectively cover a general population of young adults in an urban center.
